# Combination of Antiestrogens and Omega-3 Fatty Acids for Breast Cancer Prevention

**DOI:** 10.1155/2015/638645

**Published:** 2015-08-03

**Authors:** Andrea Manni, Karam El-Bayoumy, Christine G. Skibinski, Henry J. Thompson, Julia Santucci-Pereira, Lucas Tadeu Bidinotto, Jose Russo

**Affiliations:** ^1^Department of Medicine, Pennsylvania State University College of Medicine, Hershey, PA 17033, USA; ^2^Department of Biochemistry and Molecular Biology, Pennsylvania State University College of Medicine, Hershey, PA 17033, USA; ^3^Colorado State University, Cancer Prevention Laboratory, Collins, CO 80523, USA; ^4^The Irma H. Russo MD Breast Cancer Research Laboratory, Fox Chase Cancer Center, Temple University Health System, Philadelphia, PA 19111, USA; ^5^Barretos School of Health Sciences, Dr. Paulo Prata-FACISB, 14784-400 Barretos, SP, Brazil; ^6^Molecular Oncology Research Center, Barretos Cancer Hospital, 14784-400 Barretos, SP, Brazil

## Abstract

The molecular and biological heterogeneity of human breast cancer emphasizes the importance of a multitargeted approach for effective chemoprevention. Targeting the estrogen receptor pathway alone with the antiestrogens, Tamoxifen and Raloxifene reduces the incidence of estrogen receptor positive tumors but is ineffective against the development of hormone independent cancers. Our preclinical data indicate that the administration of omega-3 fatty acids potentiates the antitumor effects of Tamoxifen by inhibiting multiple proliferative and antiapoptotic pathways, several of which interact with estrogen receptor signaling. The complementarity in the mechanism of antitumor action of Tamoxifen and omega-3 fatty acids is well supported by our signaling, genomic, and proteomic studies. Furthermore, administration of omega-3 fatty acids allows the use of lower and, hence, likely less toxic doses of Tamoxifen. If these findings are supported in the clinical setting, the combination of omega-3 fatty acids and anteistrogens may emerge as a promising, effective, and safe chemopreventive strategy to be tested in a large multi-institutional trial using breast cancer incidence as the primary endpoint.

## 1. Efficacy and Limitations of Antiestrogens as Chemopreventive Breast Cancer Agents

Prevention represents the optimal method to reduce breast cancer morbidity and mortality. The two selective estrogen receptor modulators, Tamoxifen and Raloxifene, have been shown to be effective chemopreventive agents by reducing the incidence of estrogen receptor positive breast cancer by 50% and 38%, respectively [1, 2]. However, the wide applicability of these interventions to the population of women at large is limited by toxicity such as thromboembolic events as well as endometrial cancer in the case of Tamoxifen which, though rare, are significant when considering that these drugs are given to healthy women for prevention. The acceptance of Tamoxifen and Raloxifene for reducing breast cancer risk has indeed been shown to be poor [3]. Of the approximately 2 million U.S. women who could potentially benefit from treatment with Tamoxifen, only 4% of those at increased risk for breast cancer and only 0.08% of all U.S. women 40–79 years of age have accepted the use of this drug for chemoprevention [4–6]. A recent survey conducted in high-risk women indicates that they perceive that antiestrogens do not lower their risk of breast cancer sufficiently to justify the use of potentially toxic drugs [3]. The steroidal aromatase inhibitor exemestane has been shown to reduce the annual incidence of invasive breast cancer by 65% after a median follow-up period of three years [7]. Whether this drug will be more acceptable to the general public remains to be determined.

An additional limitation of Tamoxifen and Raloxifene is that neither drug reduces the incidence of estrogen receptor negative tumors [1, 2]. This deficiency is likely to be explained by the fact that multiple cellular pathways, in addition to the estrogen receptor, contribute to breast cancer development. Therefore, in order to optimally inhibit mammary carcinogenesis, a multitargeted approach is needed employing interventions with complementary mechanisms of action leading to increased chemopreventive efficacy and reduced toxicity. As discussed in this chapter, we believe that the addition of omega-3 fatty acids (n-3FA) to antiestrogens will increase the spectrum of molecular subtypes of breast cancer which can be prevented. In addition, we believe that this combined approach will be more acceptable in view of the perceived health benefits derived from n-3FA ingestion and the possibility of using lower and, hence, less toxic doses of antiestrogens as a result of their expected synergism with n-3FA in reducing mammary carcinogenesis.

## 2. Omega-3 Fatty Acids and Mammary Carcinogenesis

### 2.1. Epidemiological Studies

The influence of diet on breast cancer development remains controversial. The contribution to mammary carcinogenesis of the specific fatty acid composition of the diet has received considerable attention in the literature. Among the fatty acids, n-3FA and n-6FA have been suggested to decrease and increase breast cancer risk respectively [8]. Despite the perception that n-3FA protect against breast cancer, epidemiological studies have yielded inconsistent results [9, 10]. While some studies have shown an association between n-3FA intake and reduction in breast cancer risk, others have not shown this association and one has actually reported an increased risk of breast cancer with high n-3FA intake [10]. However, a recent meta-analysis of data from 21 independent prospective cohort studies revealed that dietary intake of marine n-3FA was associated with a 14% reduction in breast cancer risk [11]. Importantly, a dose-response effect was noted with a 5% lower risk of breast cancer per 0.1 gm per day increment of n-3FA intake [11].

### 2.2. Preclinical Studies

In experiments conducted both in a prepubertal [12] and postpubertal model [13] of MNU-induced rat mammary carcinogenesis, we observed that administration of fish oil providing clinically achievable ratios of n-3FA : n-6FA (up to 2.3) had a marginal antitumor action of its own and modestly influenced a variety of host and tissue biomarkers potentially involved in mammary carcinogenesis [14]. When similar clinically relevant ratios of n-3FA : n-6FA were tested in transgenic models of mammary carcinogenesis, we observed a protective effect in the HER-2neu model, a well-established model of estrogen receptor negative breast cancer (unpublished observations) in agreement with a previous report [15] but no protection in polyoma middle T transgenic mice [16]. These results suggest that gene/diet interactions play a critical role in the development of breast cancer.

**Figure 1:**
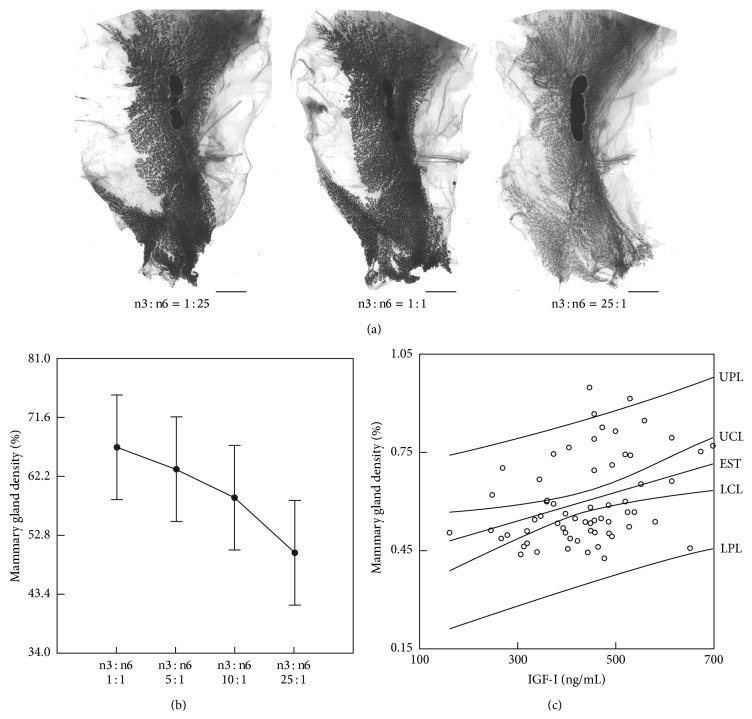
(a) Carmine-stained abdominal-inguinal mammary gland whole mounts depicting the effect of increasing dietary n-3FA : n-6FA on breast histology, bars = 0.5 cm. (b) Mammary gland density analysis shows a decreasing trend in density as n-3FA : n-6FA increases. The methodology for measurement of mammary gland density is described in detail in our publication [18]. Briefly, whole mounts of the abdominal-inguinal mammary gland chains were photographed and the images obtained were digitized. Digital images of the whole mounts were captured using a semiautomated image acquisition system. Images were evaluated for total area of the mammary fat pad occupied by mammary epithelium as well as total area of the fat pad encompassed by the mammary ductal tree. Area occupied by the mammary epithelium divided by the total area encompassed by the mammary ductal tree was calculated. (c) Linear regression of mammary gland density and IGF-I upper prediction limit (UPL); upper confidence limit (UCL); estimate (EST); lower confidence limit (LCL); lower prediction limit (LPL). Reproduced with permission from [18].

**Figure 2:**
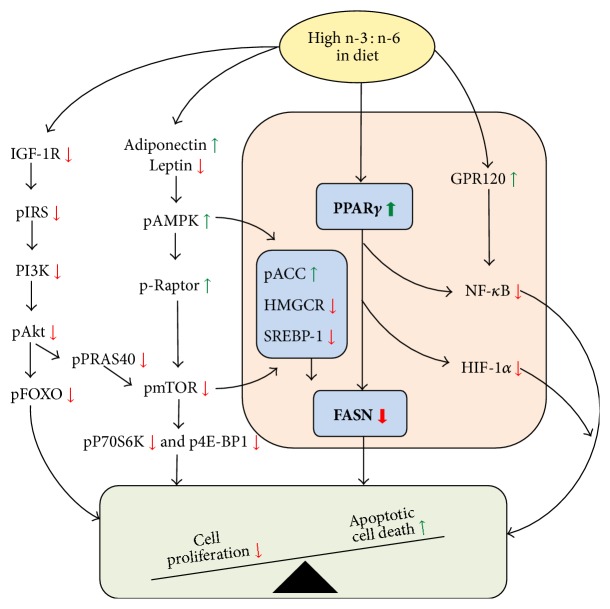
Cellular processes regulating transcription factors, insulin signaling, and lipid synthesis that are likely to account for the effects on cell proliferation and apoptosis in mammary carcinomas of rats fed high versus low (control) dietary ratio of n-3 : n-6 fatty acids. Diameter of red (decreased expression) and green arrows (increased expression) indicates magnitude of effect and font size of stated proteins indicates relative importance as determined by Orthogonal Projections to Latent Structures for Discriminant Analysis (OPLS-DA) (Refer to [21] for detailed description of the method). Peroxisome proliferator-activated receptor gamma (PPAR*γ*) and to a lesser extent, G-coupled protein receptor 120 (GPR-120) attenuate inflammation via direct or indirect effects on nuclear factor kappa B (NF*κ*B) and hypoxia-inducible factor-1*α* (HIF1*α*). PPAR*γ* affects multiple targets in lipid metabolism including fatty acid synthase (FASN). In addition, high dietary n3 : n6 is accompanied by reduced activity of the mammalian target of rapamycin (mTOR) as reflected in the reduced phosphorylation of its downstream targets including 70-kDa ribosomal protein S6 kinase (P70S6K) and eukaryotic translation initiation factor 4E-binding protein 1 (4EBP1), which in turn exert effects on cell proliferation and cell survival. Mechanisms by which mTOR activity is downregulated include (1) downregulation of insulin growth factor 1 receptor (IGF-1R), phosphorylated insulin receptor substrate 1 (pIRS1), phosphoinositide 3-kinase (PI3K), phosphorylated Akt, phosphorylated Forkhead box O, and phosphorylated 40-kDa proline-rich protein (PRAS40); and (2) upregulation of phosphorylated adenosine monophosphate-activated protein kinase (pAMPK) by increased adiponectin and decreased leptin, phosphorylated acetyl-CoA carboxylase (ACC), and phosphorylated regulatory associated protein of mTOR (Raptor). Decreased phosphorylated mTOR and increased pAMPK further attenuate fatty acids synthesis via reduction of 3-hydroxy-3-methyl-glutaryl-CoA reductase (HMGCR) and of sterol regulatory element-binding protein 1 (SREBP1) that results in decrease of FASN. The overall consequence of these changes in cell signaling is a decrease in cell proliferation and an increase in cell death by apoptosis. Reproduced with permission from [21].

These variable results prompted us to perform a critical review of the preclinical data on the role of n-3FA in mammary carcinogenesis. Our review of the literature, stemming over 30 years of investigation, produced similarly mixed results [17]. We found that the quality of the experiments varied so markedly that it was difficult to compare results across studies. In our review, a series of recommendations was made concerning the experimental approaches that would serve to guide the design of experiments with the potential of resolving the fish oil-breast cancer conundrum. These included (1) use of translationally relevant diets with 30% of calories from fat with equal distribution between monounsaturated, polyunsaturated, and saturated fats (e.g., 10% each); (2) experimental verification of FA composition of the diets in view of the multifactorial variability of n-3FA sources and bioavailability; (3) analysis of FA in the plasma and within target tissues for better comparison of results across studies; (4) consideration of variability in FA metabolism due to genetic polymorphism of related enzymes. Mindful of these issues, we decided to formulate a series of purified diets modeled after the AIN-93G formulation but with the major exception that the level of dietary fat was modified to reflect that currently recommended in the U.S. dietary guidelines. Thus, diets were formulated to provide 30% of dietary calories from fat and an equal amount of those calories from saturated, monounsaturated, and polyunsaturated fatty acids. Within the polyunsaturated fatty acids, we sought to vary the ratio of n-3FA : n-6FA from 25 : 1 to 1 : 25 to provide a robust evaluation of the role of this ratio in affecting the postinitiation phase of chemically induced mammary carcinogenesis [18]. In these experiments, at 21 days of age, female Sprague-Dawley rats were injected with 50 mg N-methyl-N-nitrosourea/kg body weight intraperitoneally. Seven days following carcinogen injection, all rats were randomized to the different diets (*n* = 30 rats/group). We observed that a calculated n-3FA : n-6FA dietary ratio of at least 10 : 1 was necessary to obtain a significant chemopreventive effect on MNU-induced mammary carcinogenesis. We also observed that increasing levels of dietary n-3FA resulted in a progressive reduction of mammary gland density (*R* = −0.477, *P* = 0.038) which was predictive of the carcinogenic response (Figures 1(a) and 1(b)) [18]. Increasing dietary amounts of n-3FA caused a significant decrease in plasma leptin and IGF-I while adiponectin levels increased [18]. However, neither cytokine was predictive of mammary gland density. In contrast, we observed a significant relationship between plasma IGF-I concentration and mammary gland density (*R* = 0.362, *P* < 0.005; Figure 1(c)) [18]. In the aggregate, these results provide evidence for the first time that breast density, a validated biomarker of breast cancer risk in women [19, 20], is a valuable screening tool for chemopreventive studies in preclinical models of breast cancer. The data also point to the importance of the IGF-I pathway in mediating the antitumor action of n-3FA. Following these observations, we performed an extensive analysis of the molecular signature underlying inhibition of mammary carcinoma by dietary n-3FA [21]. In these experiments, we analyzed tumors obtained from rats which were fed diets in which the ratio of n-3FA : n-6FA was either 0.7 (low n-3FA, control) or 14.6 (high n-3FA). We observed that cell proliferation assessed by Ki67 immunostaining was reduced by 60% in carcinomas from the high n-3FA : n-6FA (14.6 ratio) treatment group and was associated with a reduction in the levels of cyclin-D1 and phospho-Rb as well as an increase in the levels of two cyclin dependent kinase inhibitors, p21 and p27, as determined by Western blotting and densitometric analysis. These changes are consistent with a block at the G1-S transition induced by the high n-3FA diet. The apoptotic index (computed as the number of apoptotic cells divided by the total number of cells counted) was significantly increased by 29% in carcinomas from the high n-3FA : n-6FA group. Relative to apoptosis and consistent with the elevated apoptotic index observed in the high n-3FA diet group, the level of cleaved PARP (PARP89/116 ratio) was elevated as were levels of Bax and Apaf-1, whereas the level of Bcl-2 was not significantly affected. These changes are indicative of the induction of apoptosis via the intrinsic pathway. The suppressive effect of n-3FA on proliferation was dominant over its effect on induction of apoptosis. Using Western blotting followed by densitometry, we performed an extensive analysis of transcription factors, growth factor-related molecules and proteins involved in lipid metabolism in the attempt to identify the cellular mechanisms by which a high n-3FA diet leads to inhibition of proliferation and induction of apoptosis. The results are summarized in Figure 2 [21]. As described in detail in the figure legend, the predominant effect of high n-3FA diet was PPAR*γ* activation resulting in suppression of lipogenesis primarily through downregulation of fatty acid synthase (FASN). In addition, high n-3FA diet suppressed mTOR pathway (well established to be critical in carcinogenesis) both by suppressing IGF-I signaling and upregulating pAMPK as a result of the reduction in leptin and the increase in adiponectin. Furthermore, the activation of pAMPK also contributed to the inhibition of lipogenesis through its effect on key regulators of lipid synthesis (pACC, HMGCR, and SREBP-1), thus potentiating the effect of PPAR*γ* activation on this critical metabolic parameter. The fact that high ratios of n-3FA : n-6FA were required to achieve profound antitumor effects not only indicates that these biological activities are not likely to be achieved by dietary consumption of fish oil but also that there are specific metabolites of n-3FA that account for these effects and that are likely to be endogenously synthesized. The identification of these metabolites is currently under active investigation in our laboratories.

## 3. Combination of Omega-3 Fatty Acids and Antiestrogens

A major focus of research in our laboratories has been to test the antitumor efficacy and safety of the combination of n-3FA and antiestrogens for breast cancer prevention. The rationale behind this approach is based on the multiplicity of signaling pathways affected by omega-3 fatty acids (Figure 2), several of which are well known to interact with the estrogen receptor pathway [21]. For instance, there is a well-documented crosstalk between the estrogen receptor and the PPAR*γ* receptor [22, 23], the latter being a major mediator of n-3FA effects in breast cancer cells. There is experimental evidence that inhibition of estrogen receptors with antiestrogens and activation of PPAR*γ* synergistically downregulates the PI-3 kinase/AKT pathway and inhibits breast cancer cell proliferation [22]. Because of the complementarity of their antitumor action and the known antiproliferative effects of n-3FA in estrogen receptor negative breast cancer cell lines [15, 16, 24], we believe that the chemopreventive effect of the combination of n-3FA and antiestrogens will not be restricted to ER positive tumors but will extend to ER negative tumors which are more aggressive and associated with shorter survival.

### 3.1. Preclinical Studies

In experiments conducted both in a prepubertal and postpubertal model of MNU-induced rat mammary carcinogenesis, we observed that administration of fish oil providing clinically achievable ratios of n-3FA : n-6FA (up to 2.3) potentiated the chemopreventive effects of Tamoxifen [12, 13]. The potential superiority of the combination was particularly evident at a suboptimal dose of Tamoxifen which, by itself, was unable to significantly decrease tumor development [12]. We recently reported a detailed time course study of the individual and combined chemopreventive effects of Tamoxifen and a high fish oil diet on multiple histologic parameters of MNU-induced mammary carcinogenesis [13]. In these experiments, groups of female Sprague-Dawley rats were injected ip with MNU at 50 days of age and randomized to either a control diet (20% corn oil) or a fish oil (FO) rich diet (10% FO + 10% CO) with or without the addition of Tamoxifen (Tam) in the diet (0.6 ppm). Rats (18/group) were sacrificed at weeks 4 (before palpable tumors), 8, and 12 (when ~90% of control rats had palpable tumors). In addition to removing palpable tumors, abdominal-inguinal mammary fat pads were excised for full histologic analysis of preneoplastic lesions classified as mild hyperplasia, modest hyperplasia, and florid hyperplasia as well as ductal carcinomas* in situ* and invasive adenocarcinomas. Our results indicated that the FO rich diet enhanced the antitumor action of Tam on all histologic parameters of carcinogenesis. Importantly, we observed that the combination treatment was the only intervention that not only inhibited the development of preneoplastic lesions but also induced regression of established ones. This effect is of particular translational relevance since it is likely that when a chemopreventive intervention is applied to women, preneoplastic lesions are already present.

**Figure 3:**
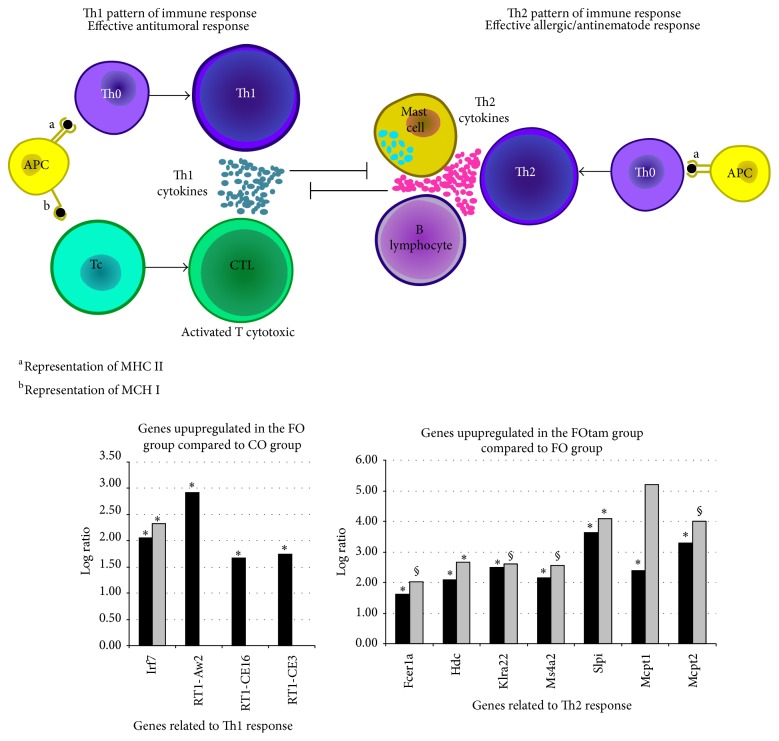
Diagram depicting the patterns of immune responses found represented among the genes upregulated by fish oil (FO) and Tamoxifen in a FO rich diet (FOtam). Graphs show side-by-side log 2 values of gene expression in microarray (black bars) and real time PCR (grey bars) of genes related to the immune response. ^*^ *P* < 0.05; § 0.05 < *P* < 0.20, with fold change >3.0 (log 2 > 1.58). Figure modified from [25].

**Figure 4:**
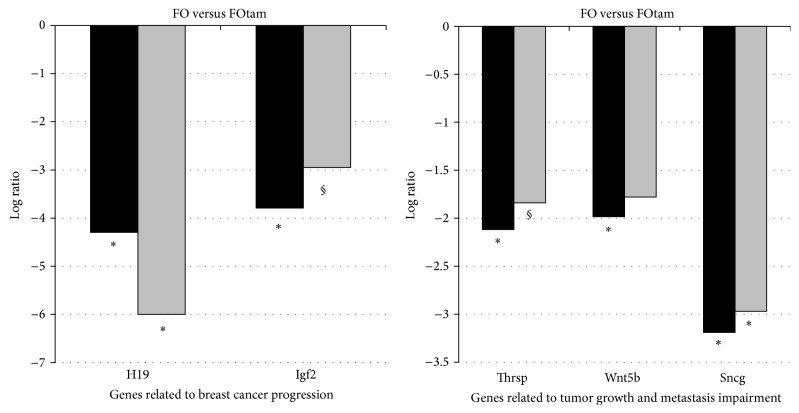
Side-by-side log 2 values of gene expression in microarray (black bars) and real time PCR (grey bars) of genes related to tumor profile. ^*^ *P* < 0.05; § 0.05 < *P* < 0.20, with fold change >3.0 (log 2 > 1.58). Figure adapted from [25].

To gain insight into the potential mechanisms underlying the superior chemopreventive efficacy of the combination, we have performed transcriptomic analysis (microarray followed by real time PCR validation of select genes of interest) in the tumors of control rats and Tamoxifen-treated rats each fed either a corn oil or fish oil rich diet [25]. We used gene ontology analysis and analysis of the relation of each differentially expressed gene with cancer related processes. We identified alterations in genes directly related to the biologic features of breast cancer (such as tumor differentiation and progression) as well as genes related to the immune response. Gene ontology enrichment analysis showed that administration of a fish oil rich diet resulted in the differential expression of several genes that promote a more efficient immune response against tumor development (Figure 3). In addition, tumors of fish oil fed animals that received Tamoxifen showed decreased mRNA for genes directly related to tumor growth and metastasis (Figure 4), thus indicating that Tamoxifen treatment was more efficient in a fish oil rather than corn oil diet background. On the other hand, we observed that the expression of genes associated with immunity in animals in the fish oil + Tamoxifen group indicated a shift to the Th2 pattern of immune response which may favor tumor escape (Figure 3) [25]. In conclusion, a FO rich diet resulted in the differential expression of several mRNAs that encode genes that promote more differentiated tumors and a more efficient immune response against tumorigenesis compared to a CO rich diet. While genes related to tumor growth and metastasis were downregulated by Tamoxifen in FO fed rats, our data also point to a potential immunologic mechanism of tumor escape from the combined intervention.

**Figure 5:**
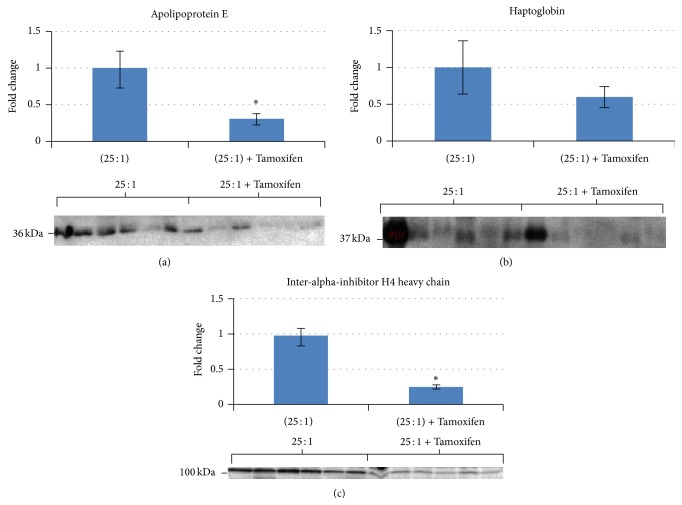
Western blot analysis of specific proteins for validation of iTRAQ analysis (comparison of 25 : 1 n-3 : n-6 with 25 : 1 n-3 : n-6 plus Tamoxifen). (a) Lipoprotein E expression; (b) haptoglobin expression; (c) inter-*α*-inhibitor H4 heavy chain expression; ^*^ *P* ≤ 0.05. Reproduced with permission from [26].

We have also used a proteomic approach to gain insights into the mechanism of protection at the protein level by n-3FA in the absence and in the presence of Tamoxifen [26]. Using the isobaric tags for relative and absolute quantitation (iTRAQ) followed by confirmation by western blots, we found that increasing ratios of n-3FA : n-6FA in the diet induced dose-dependent changes in the plasma level of several proteins in a manner consistent with chemoprevention. Those included an increase in gelsolin and vitamin D binding protein, both shown to have tumor protective properties [27, 28]. A high ratio of n-3FA : n-6FA also increased the expression of 14-3-3 sigma, a well-known tumor suppressor gene [29]. In contrast, alpha-1*β*-glycoprotein, shown to be increased in a variety of cancers [30–32] was reduced by a high n-3FA diet. We also observed that the combined administration of Tamoxifen with a high ratio of n-3FA : n-6FA altered additional proteins also in a manner consistent with chemoprevention (Figure 5) [26]. These changes included a reduction in apolipoprotein E, haptoglobin, and inter-alpha inhibitor H4 heavy chain all shown to have tumor promoting properties [33–35]. Measurement of these differentially regulated proteins could be useful for monitoring the efficacy of n-3FA and Tamoxifen as chemopreventive agents in clinical trials.

### 3.2. Clinical Studies

**Figure 6:**
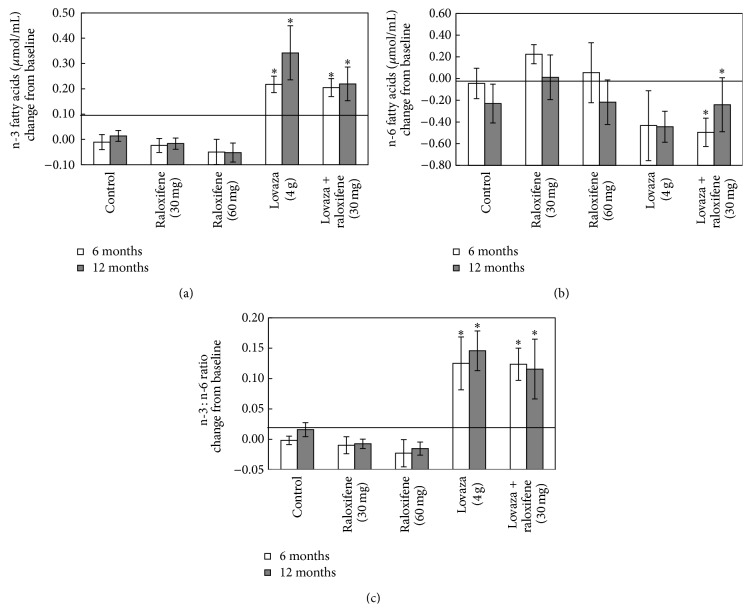
Treatment effects on serum n-3FA (a), n-6FA (b), and their ratio n3FA/n-6FA (c). The number of subjects was 8 in the control, 11 in the Lovaza 4 g group, and 8 in the Lovaza 4 g + Ral 30 mg group. Data represent mean values ± s.e.m. ^*^ *P* < 0.05 versus the other groups. Reproduced with permission from [36].

While basic mechanisms of cooperativity between n-3FA and antiestrogens are under investigation using preclinical models of mammary carcinogenesis, we are testing concomitantly the clinical relevance of this approach in postmenopausal women using a reduction in breast density, a well-established risk factor for breast cancer [19, 20], as our primary endpoint. We have just completed a clinical trial involving 266 healthy, postmenopausal women at increased risk for breast cancer based on a breast density ≥25%, detected at their annual screening mammogram. They were randomly assigned to one of the following groups: (1) no intervention; (2) Raloxifene 60 mg; (3) Raloxifene 30 mg; (4) omega-3 fatty acids (Lovaza) 4 gm; and (5) Lovaza 4 gm + Raloxifene 30 mg for two years (NCT00723398). While the final analysis of the effects of our interventions on breast density is in progress, we have published a preliminary report demonstrating the feasibility and acceptability of this approach with excellent compliance (96 ± 1% overall) by pill count [36]. In addition, we found that Lovaza administration increased the plasma levels of n-3FA by about 3-fold (Figure 6); such levels are quite comparable to those we have reported to exert chemopreventive effects in rats fed fish oil diet [12, 14], thus supporting the translational relevance of our animal studies described above.

## 4. Concluding Remarks

Despite the efficacy of antiestrogens in breast cancer prevention, there is an urgent need to develop safer and more effective chemopreventive strategies which could also inhibit the development of estrogen receptor negative tumors which are more aggressive and associated with a shorter survival. We judge that a combination approach targeting several cellular pathways involved in mammary carcinogenesis is needed for optimal antitumor efficacy. Our preliminary data strongly suggest that the combination of n-3FA and antiestrogens is superior to the individual interventions in reducing the incidence and multiplicity of chemically induced mammary tumors in rats. Our signaling, genomic, and proteomic studies suggest complementarity in the mechanism of antitumor action of antiestrogens and n-3FA which allows the use of lower and hence less toxic doses of antiestrogens without losing chemopreventive efficacy. The clinical relevance of these observations will soon be revealed in the final analysis of our recently completed clinical trial where we use a reduction in breast density, a well-established biomarker of breast cancer risk, in assessing the antitumor effects of our intervention. If positive, the results of our study would provide the rationale for launching a large multi-institutional trial testing the chemopreventive efficacy of n-3FA and antiestrogens using breast cancer incidence as the primary endpoint.
